# Image-Based Feedback of Multi-Component Microdroplets for Ultra-Monodispersed Library Preparation

**DOI:** 10.3390/mi15010027

**Published:** 2023-12-22

**Authors:** Christy Cantwell, John S. McGrath, Clive A. Smith, Graeme Whyte

**Affiliations:** 1 Institute of Biochemistry, Biophysics and Bioengineering, Heriot-Watt University, Edinburgh EH14 4AS, UK; 2 Sphere Fluidics Limited, Granta Centre, Granta Park, Great Abington, Cambridge CB21 6AL, UK

**Keywords:** microdroplets, microfluidics, image analysis, microscopy, feedback

## Abstract

Using devices with microfluidic channels can allow for precise control over liquids flowing through them. Merging flows of immiscible liquids can create emulsions with highly monodispersed microdroplets within a carrier liquid, which are ideal for miniaturised reaction vessels which can be generated with a high throughput of tens of thousands of droplets per second. Control of the size and composition of these droplets is generally performed by controlling the pumping system pushing the liquids into the device; however, this is an indirect manipulation and inadequate if absolute precision is required in the size or composition of the droplets. In this work, we extend the previous development of image-based closed-loop feedback control over microdroplet generation to allow for the control of not only the size of droplets but also the composition by merging two aqueous flows. The feedback allows direct control over the desired parameters of volume and ratio of the two components over a wide range of ratios and outperforms current techniques in terms of monodispersity in volume and composition. This technique is ideal for situations where precise control over droplets is critical, or where a library of droplets of different concentrations but the same volume is required.

## 1. Introduction

The generation of miniature drops within the controlled environment of microfluidics, so-called microdroplets in microfluidics, provides a powerful platform for developing microscale reaction vessels for chemical [1,2] and biochemical reactions [3,4], library generation [5,6] and screening, and nanoparticle fabrication [7], amongst many other applications. Recent reports now indicate that reaction kinetics are increased and accelerated under microconfinement, notably in droplets, thin films, and emulsions [8,9]. The relatively straightforward process of combining two immiscible phases within a microfluidic channel geometry can generate microdroplets of one phase dispersed within the other. The geometry of the channels and additives such as surfactants and wall coatings control which is the dispersed phase and which is the continuous phase, and their flow rates control the size and rate of microdroplet generation [10]. In many applications, this provides enough control; however, where additional precision and/or accuracy of droplet size and composition is needed, it can often be a very time-consuming through a trial-and-error approach to determine the correct parameters needed to generate the required droplets. The accurate control of droplet volume is critical in many applications of microdroplets where changes in the volume give rise to errors in the estimation of the concentration of a product, or when encapsulating single objects, e.g., single molecules [11] or cells [5,12], or even approximately one hundred cells per droplet [13], where Poisson statistics [14] is crucially important.

Syringe pumps are a very common tool for microfluidic research as they offer a straightforward method to infuse a given flow rate into a microfluidic device. As the motor moves, the pressure is increased as required to flow at the given flow rate and maintains the given average flow rate without intervention, unless it reaches the stall force of the motor. This makes them ideal for continuous-flow experiments where a set of constant flow rates are required; however, for systems such as microdroplet experiments, the absolute flow rates are often not the most meaningful of parameters, with droplet volume, droplet-to-oil fraction, and droplet generation frequency, for example, being much more intuitive and relevant parameters to consider and control. These parameters are not direct inputs into an experiment but a complex interplay of the physical properties of the phases, the channel geometry, and the driving flow rates, and so the direct control of these parameters is not possible. However, the use of image-based feedback can provide control over these parameters using feedback loops to measure the desirable parameter(s) and modify the controlling parameters in response to changes [15,16,17]. The use of image analysis to not simply measure but also control aspects of microdroplets and microfluidics has expanded dramatically recently with the improvement in camera framerates, microsecond exposure times allowing real-time image-processing algorithms, and the implementation of machine learning approaches, which even allow for droplet sorting based on image analysis [18,19,20,21,22,23].

In this study, the use of image-based measurement of microdroplets for closed-loop feedback control is extended beyond the state of the art to allow the controlled generation of two-component droplets, where the user can define the desired physical volume of the droplets and the relative concentration of the two components. 

Current techniques have been limited to producing ultra-monodispersed microdroplets from a single aqueous inlet stream, limiting the potential uses of the technology [1]. By extending image-based feedback to a dual-aqueous system, droplets of two components can be created and the relative ratio between them controlled. Building upon image-based droplet volume measurement techniques, which can provide a droplet-by-droplet measurement of the volume combined with frequency determination from the analysis of the droplet light scattering on a photodetector, the total aqueous flow rate can be calculated. If a capillary-based flow meter is used within one of the two aqueous input lines, the flow rate of the other can be inferred. By using only a single flow meter, it reduces the overall system complexity and has many advantages in allowing one of the two components to have a smaller volume requirement, minimising cross-contamination issues, fouling, or blockage in the capillary-based flow meter, especially in preventing damage to samples containing particles or cells. 

The extension of feedback techniques to multi-component droplets allows ultra-monodispersed generation in more complex situations and would be highly beneficial wherever accurate control of the volume and concentration is required, for example in chemical and biochemical assays, particularly involving single-molecule or single-cell studies, where a small change in the volume or dilution ratio can have a large effect on the results. It is also highly beneficial for the generation of libraries of droplets with different components but the same tightly controlled volume, which would be beneficial in screening applications where a wide range of accurately known concentrations should be analysed in a high-throughput manner, for example in pharmacological or toxicological studies.

## 2. Materials and Methods

### 2.1. Experimental Setup

**Figure 1:**
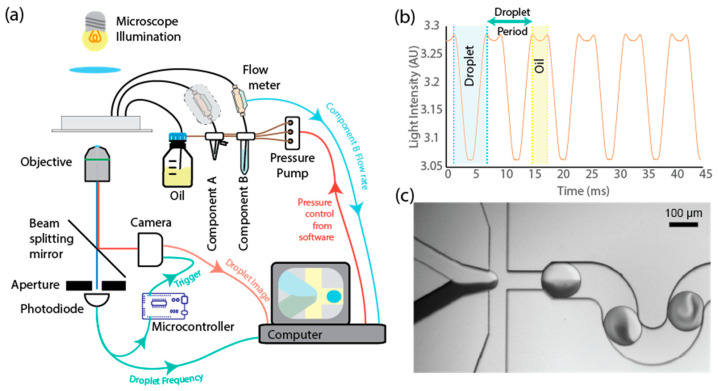
(**a**) Schematic showing the experimental setup consisting of a microscope equipped with a triggered camera and droplet detector (photodiode) used to image the droplets in flow through the microfluidic device, and a fluid pumping system of air-over-liquid pumps controlled by software to produce droplets of a given size and composition. Coloured arrows represent the flow of data, with the arrow depicting the source of data pointing to the destination. (**b**) Example photodiode voltage trace showing the passing of 5 droplets over the detector region. Analysis of the trace provides the frequency of droplet generation used in the feedback calculation. (**c**) Micrograph of the droplet generation junction showing the meeting of two aqueous components (PBS—top; Trypan blue dye—bottom) before droplet generation. Scale bar is 100 µm.

The experimental setup was arranged as shown schematically in Figure 1 and built around a custom microscope. The microscope consisted of a 25× microscope objective (Fisherbrand, Loughborough, UK, 25×/0.4 Long Working Distance) with a 100 mm tube lens (Thorlabs AC254-100) and imaged onto a camera (Basler, Ahrensburg, Germany, ACE 270 gm). Illumination was supplied through a white-light LED (4000 K, CreeLED, Durham, NC, USA, CXB18300N0BV240E) and custom-built condenser. The camera supports an “ultra short exposure” mode of 1 µs to minimise motion blur when imaging moving microdroplets. 

To aid in the image analysis and frequency estimation, the light from the objective was split by a dichroic mirror (Semrock, Rochester, NY, USA, Di03-R488), passed through a linear slit aperture (Thorlabs Ltd., Ely, UK, VA100CP/M), and detected by an amplified photodiode (Thorlabs Ltd., Ely, UK, PDA36A2). As the droplet moves through the channel, the change in intensity can be detected on the photodiode at high speeds using data acquisition hardware. This allows accurate frequency estimation beyond the framerate of the camera to ensure no droplet is missed by the camera acquisition time. The photodiode signal is additionally processed by a microcontroller running a custom script to detect the leading edge of microdroplets. When the leading edge is detected, a trigger signal is sent to the camera to ensure that each frame captured has a droplet centred in the image.

The input fluidics were driven by a pressure controller (Elveflow 200mbar OB1, Elvesys, Paris, France), which was directed by a PC running National Instruments LabView 2021. One of the two input fluids was passed through a capillary-based flow meter (LG16-0150D, Sensirion AG, Stäfa, Switzerland) to monitor the flow rate of that component. To validate this proof of concept, the second fluid also passed through a flow meter; however, the measured flow rate was not used as part of the feedback and was solely for the validation of the technique. By only requiring a single flow meter, the system would allow precious samples to be used, which may not be compatible with flowing through a flow meter, either due to the large swept volume, wishing to minimise contamination, or because the samples are laden with particles or cells, which may be damaged by passing through the capillary. 

#### 2.1.1. Device Fabrication

The devices were fabricated from PDMS using the widely used method of photolithographically defined SU-8 on a silicon master, as described elsewhere [24,25]. Briefly, SU-8 masters were produced by spin coating Si wafers with SU-8 (SU-8 2025 MicroChem) and exposed to UV light through a mask to initiate cross-linking, which was enhanced by baking. The non-cross-linked SU-8 was removed using developer solution (PGMEA, MicroChem Inc., Newton, MA, USA), leaving a mould which can be used to produce multiple identical devices. Liquid PDMS (Sylgard 184, Dow Midland, MI, USA) mixed in a 10:1 elastomer/curing agent ratio was poured onto the master and baked in a 60 °C oven for 3 h before being cut out and access holes punched using a biopsy puncher. The device and PDMS base were treated with an air plasma apparatus (Zepto, Diener, Ebhausen, Germany) and brought into contact to seal. Then, they were placed in an oven at 110 °C for at least fifteen minutes. On cooling to room temperature, the inner surfaces of the devices were treated with PicoGlide (Sphere Fluidics, Cambridge, UK) by flushing the solution into the channels, which was ideally left overnight and then cleared with compressed air to render the surfaces hydrophobic. 

#### 2.1.2. Device Operation

Devices were mounted onto the custom-built microscope and fluids infused using two different pumping methods. Syringe pump data were obtained using stepper motor pumps (KD Scientific, Holliston, MA, USA, Legato^®^ 130) with 1 mL plastic syringes (BD Norm-ject luer lock) with blunt needles. Pressure over liquid pumping was achieved using the pressure regulator described above. 

To easily visualise the two components and the relative concentration within the droplets, a mixture of Trypan blue (Merck, Dorset, UK) and Phosphate Buffer Solution (PBS) was used. The Trypan blue absorbed the illumination light from the microscope and showed up as a dark intensity in the camera images, while the PBS was transparent, resulting in an intensity similar to the background. As can be seen in Figure 1, the two components can be clearly visualised. It is important to note that the use of an absorbing dye is not required for the operation of the system.

The triggered camera images were captured for every droplet at a droplet generation rate of up to ~110 Hz, beyond which the processing and saving time for each image caused the capture rate to be limited. Capturing an image of each droplet is not required for the feedback to function as the photodiode is used to measure the droplet frequency, which can exceed the capture rate of the camera. To ensure that the droplets were well mixed and the interface between the two components did not affect the measurement of the volume, images were acquired after the serpentine channel, which increased the rate of mixing within the droplet [26,27]. This can be avoided in cases where the two components have similar optical properties. The photodiode data were continuously captured using a data acquisition card (National Instruments Austin, TX, USA, USB-6211). The flow meter values were read continuously at the sample rate of the flow meters (200 ms).

#### 2.1.3. Software Setup

Control over the droplet volume and composition was achieved using bespoke LabView Virtual Instruments to acquire the data, process them, and regulate the pressure pumps. The software captured the triggered camera image and calculated the currently generated droplet volume from the linear length of the droplet and knowledge of the channel geometry. The calculation was performed using the three equations corresponding to droplets of different geometries [28]:

$$V=\frac{4}{3}\pi {\left(\frac{l}{2}\right)}^{3}\mathrm{when}\;l<W\;\mathrm{and}\;H$$

$$V=\frac{\pi }{3}\left(4{\left(\frac{l}{2}\right)}^{3}+3\pi {\left(\frac{l}{2}\right)}^{2}+6\frac{l}{2}{\left(\frac{W-H}{2}\right)}^{2}\right)\;\mathrm{When}\;W>l>H$$

$$\;V=\left(HW-\left(4-\pi \right){\left(\frac{2}{H}+\frac{2}{W}\right)}^{-2}\right)\left(l-\frac{W}{3}\right)\;\mathrm{When}\;l>W\;\mathrm{and}\;H\;$$

The droplet generation frequency $f_{gen}$ was calculated from the photodiode signal using a Fourier transform-based tone extraction process. The total dispersed volumetric flow rate can thus be calculated as $Q_{dis}={V\cdot f}_{gen}$. The measured flow rate of component B, $Q_{B}$, can then be subtracted from this to give the flowrate of component A, $Q_{A}=Q_{dis}-Q_{B}$ and the concentration, *C*, calculated as ${C=Q}_{B}/Q_{dis}$.

The feedback system monitored the measured droplet volume and concentration and compared these to the target values. It updated the pressures acting on the pumps using a feedback routine:

$$C_{N+1}=C_{N}+k_{c}\left(C_{N}-C_{target}\right)\;p_{dis\;N+1}=p_{dis\;N}+k_{V}(V-V_{target})$$

$$\begin{array}{c} p_{1}=const.\;\;\\ p_{2}=c_{N+1}\cdot p_{dis\;N+1}\\ p_{3}=\left(1-C_{N+1}\right)P_{dis\;N+1\;} \end{array}$$

where $C_{N+1}$, $C_{N}$, and $C_{target}\;$ are the measured concentration for the current iteration, the previous iteration, and the target concentration, respectively. $p_{dis\;N+1}$ and $p_{dis\;N}$ are the total dispersed pressure for the current and previous iteration. V and $V_{target}\;$ are the measured droplet volume and target droplet volume. $k_{C}$ and $k_{V}$ are the feedback coefficients for the concentration and pressure, respectively, and $p_{1},\;p_{2}\;\mathrm{and}\;p_{3}$ are the pressures sent to the continuous phase, component B, and component A, respectively. $k_{C}$ and $k_{V}$ define how quickly the controlling pressures change in response to a change in the measured parameters. A lower k value results in a slower response but is less susceptible to variations due to noise. Raising the k value can increase the responsiveness of the system, but this can cause the system to respond to small fluctuations due to measurement noise, which can decrease the monodispersity in volume or concentration. If the k value becomes too high, it can cause the system to dramatically change the pressure, which can result in the system overshooting the target value as well as cause instability and additional oscillations. The value of the k coefficients are dependent on a complex mix of the time response of the overall system, the fluidic resistance of the device which determines the flow rate change for a given pressure change, as well as the noise present and the desired responsiveness. By changing the relative value of the k coefficients, the stability of one parameter can be prioritised over the other; generally, it is envisioned that $k_{V}$ will be larger than $k_{C}$, which results in the volume feedback being more responsive than the concentration feedback to maintain the target volume, even when the target concentration is altered.

## 3. Results and Discussion

**Figure 2:**
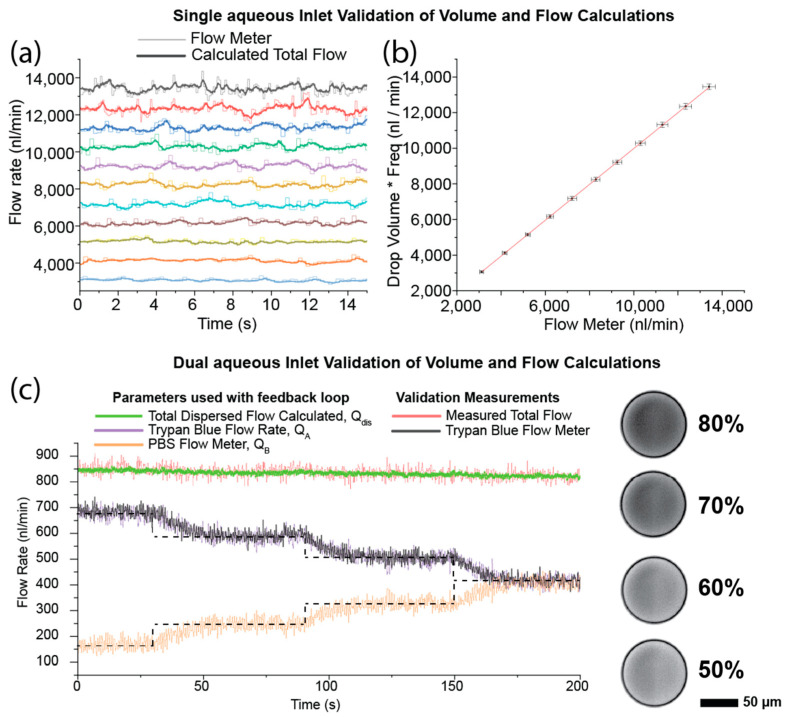
Validation of the droplet volume measurement and flow rate calculation. (**a**) Comparison of the estimated flow rate based on the droplet volume and frequency measurements, and the measured flow rate for a single aqueous inlet experiment across a range of flow rates. Thick lines show the measurement used for the feedback loop; thin lines show the measured flow rate, colours represent different driving pumping rates. (**b**) The data from (**a**) showing the estimated flow rate against the measured flow rate. Error bars are the standard deviation in flow rate. Droplet generation rate was ~100 Hz. (**c**) Validation of the flow measurements in a dual-aqueous experiment. The graph shows the parameters used in the feedback loop compared to the values obtained using the additional validation flow meter. Excellent agreement between the two measurements can be seen. Inset shows example droplets containing a mixture of Trypan blue dye and PBS at different stages of the experiment. Dotted lines denote the target flow rates as the desired concentration was altered. Scale bars correspond to 50 µm.

To ensure that the experimental assumptions, droplet volume and frequency measurements, and the resulting flow rate calculation are valid, the results of validation experiments using only a single aqueous inlet of the device are shown in Figure 2a,b. By sealing the second inlet of the device, the measured flow rate can be compared with the calculation from the droplet size and frequency. If the measurement and calculations are correct, the estimated flow rate should agree with the measured flow rate across a wide range of flow rates. As can be seen in Figure 2a,b, there is good agreement between the calculated and measured flow rate values, giving confidence that the measurements are accurate and the technique viable. Figure 2c further extends this confirmation by using the validation flow meter (the outlined flow meter on component A in the schematic in Figure 1a) to compare the calculated flow rate for component A (Figure 2c) to the measured flow rate. It should be stressed that the validation flow meter was not used in the feedback calculation and would not be part of the proposed final setup beyond testing and validation. By removing this flow meter, the swept volume of the tubing for component A can be made significantly smaller, or removed entirely by using an on-chip reservoir, allowing precious samples to be efficiently encapsulated. The estimation of the total flow rate shows reduced noise compared to the addition of the two flow meter values; this is due to the slower sampling rate and discretisation noise of the flow meters, resulting in their uncertainties combining to produce a less precise result. Images of the microdroplets produced as a mixture of Trypan blue dye and Phosphate Buffer Solution (PBS) show a clear change in the intensity of the droplets with the changing concentration of dye, as would be expected. 

**Figure 3:**
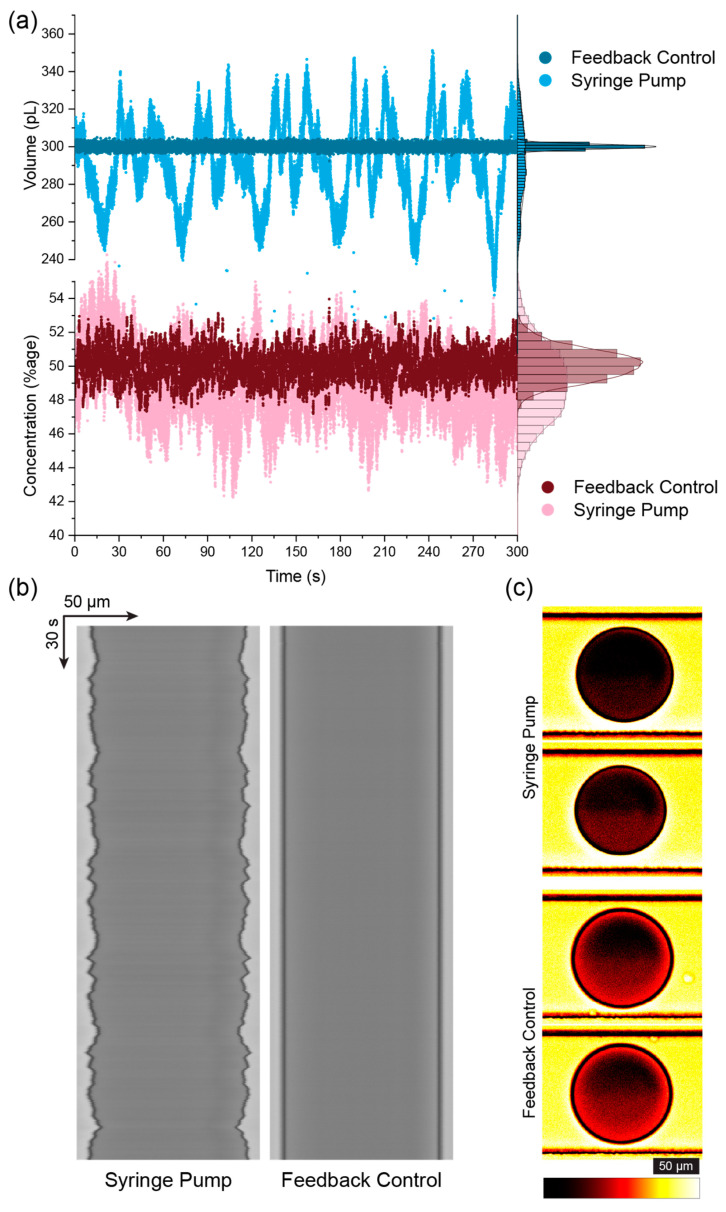
(**a**) Comparison of the droplet volume (top, blue) and composition (bottom, red) obtained when using the developed system (dark blue, dark red) and using a standard syringe pump (light blue, light red). Scatter plot shows individual droplet measurements over time, while the histograms on the right show the normalised distributions. Significant improvements can be seen in both the volume and composition using the image-based feedback approach. (**b**) Kymographs of the midline of the droplet over time comparing the syringe pump (left) to the image-based feedback (right) for droplets composed of ~50% Trypan blue dye and 50% PBS. (**c**) Contrast-enhanced images of the droplets showing the variation in measured intensity between the highest and lowest measured droplet concentration for the syringe pumps (top) and image-based feedback (bottom). All images in (**c**) were processed with the same adjustment levels and look up table. Scale bars correspond to 50 µm.

To demonstrate the advantages of feedback control over existing dual-aqueous droplet techniques, similar droplets were created using traditional syringe pump infusion and the feedback method. As can be seen in Figure 3, the use of the developed system improved the quality of the droplets composed of two aqueous input streams, both in terms of the overall droplet volume and the droplet concentration. The stepper motor-based syringe pump led to oscillations in the flow and pressure, resulting in time-dependent oscillations in the droplets, as can be seen in Figure 3a,b. The improvements in droplet volume control were previously shown and are of a similar value to previously published data [15]. The results for the droplet concentration also show an improvement for the feedback system, although the concentration for the syringe pump is surprisingly consistent given the large variability in droplet sizes and lack of synchronisation between the pumps.

**Figure 4:**
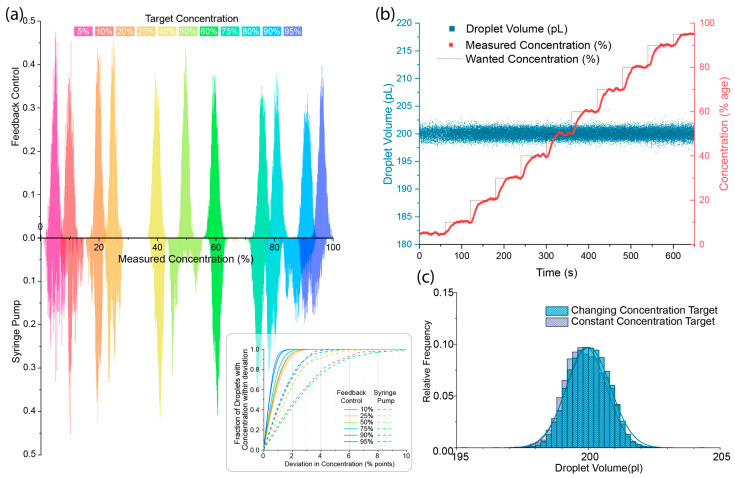
(**a**) Histogram distributions of the measured droplet concentration for a range of target concentrations obtained using the image-based feedback approach (top) and syringe pumps (bottom). Inset shows the cumulative distribution of droplets which have a concentration within a given concentration range. The image-based feedback approach (solid lines) shows a large improvement in the droplet distribution, with 99% of droplets being within 2.8 percentage points of the target, and the syringe pumps achieved 99% of droplets within 9.6 percentage points. (**b**) Scatter plot of the droplet volume (blue) and concentrations (red) when changing the target concentration over a range from 5% to 95% (red solid line) showing a consistent droplet volume is maintained, even when changing concentration. Droplet generation rate was ~30 Hz. (**c**) Histograms of the droplet volume obtained in (**b**) while changing droplet volume (blue) and comparable data obtained for a constant target concentration (purple) showing the consistent volume distribution.

Since the feedback system continuously adjusts to maintain a target value, the droplet volume and/or concentration can be changed interactively to produce a series of droplets with different properties. Figure 4 shows how the system performs at different target concentrations. Figure 4a shows the improvements in droplet concentration control are not limited to the 50:50 case already shown in Figure 3, and as the relative flow of the two input streams diverge, the feedback system outperforms the stepper motor syringe pump further. By altering how quickly the feedback loop adjusts the input pressures, it is possible to prioritise different aspects of the feedback control, as can be seen in Figure 4b, where the droplet volume is prioritised over concentration, leading to a slower reaction to changes in the target concentration but resulting in the droplet volume remaining consistent. The coefficient of variation during the change from 5:95 to 95:5 concentrations remained virtually unchanged compared to the coefficient of variation for a constant 50:50 target over a similar time range (Figure 4c). This allows the system to generate libraries of highly monodisperse droplets of the same volume but with varying concentrations.

**Figure 5:**
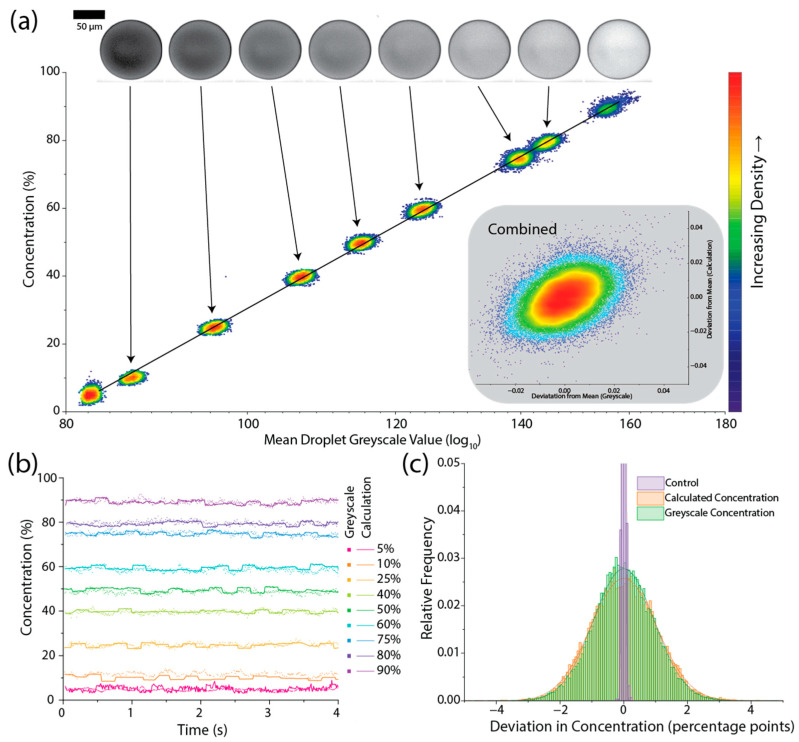
(**a**) Scatter heatmaps of the measured droplet concentration obtained using the flow meters compared to the greyscale value obtained from the droplet image, showing the strong correlation between the two measurements. Inset shows the combined deviation from the mean for all droplets. (**b**) Concentration measurements over a short time period showing the correlation between the greyscale measurement of each droplet (square points) and the measurement used in the feedback process (solid line) for a range of concentrations. (**c**) Histograms of the deviation in concentration for the calculation used in the feedback (orange) and the greyscale measurement (green), as well as a control sample consisting of droplets generated from a single inlet of 50% Trypan blue and PBS prepared off-chip (purple). Note the purple control histogram is cropped for clarity. Scale bar corresponds to 50 µm.

In all cases so far, the estimated droplet concentration was based on a flow meter measurement with an integration time of ~200 ms, which is significantly slower than the rate of droplet production of ~100 Hz. This may lead to an underestimation of the concentration variability due to the multi-droplet integration time. To test if the estimated concentration was representative of the droplet-to-droplet variability and ensure the measured concentration is accurate, validation was carried out using the concentration-dependent absorption of a dye within the droplets. Figure 5a shows the correlation between the greyscale intensity (shown on a log_10_ scale) and the fraction of PBS within the droplets. Across the ranges tested, there was a clear correlation, which can be seen in the trends between the target concentrations but also from the distribution within each target concentration shown by the diagonal distribution of the scatter. By calculating the concentration from the greyscale measurement, the droplet-to-droplet variability can be compared to the estimated concentration measurements based on the droplet volume estimation, as shown in Figure 5b. There is strong agreement between the two measurements, and there was no significant increase in the spread of concentration measurements when the concentration of each droplet was measured more directly (Figure 5c). For reference, the concentration measurement from the greyscale image of a 50:50 mix of Trypan blue and PBS prepared off-chip and injected into a single inlet chip is shown, truncated for clarity, in Figure 5c, showing the distribution of droplet concentration is not limited by measurement error in the intensity measurement and is representative of the variation in concentration within the droplets.

## 4. Conclusions

In conclusion, the presented image-based feedback technique represents a significant step forward in the control of microdroplet generation, allowing the accurate control of droplet composition as well as droplet volume. The use of physical parameters of interest (droplet volume, composition, and frequency), rather than flow rates or pressure, allows non-experts to easily create droplets with the appropriate requirements, and the feedback provides reassurance that they are of the correct properties and do not drift over time. The use of two inlets dramatically increases the range of experiments for which image-based feedback can be used. The ability to maintain a given droplet volume despite changing the composition of the droplets over time opens the door to the generation of libraries of ultra-monodisperse droplets with a range of precisely defined concentrations in a single experimental run.

## Data Availability

Data are available upon reasonable request from the corresponding author.
